# Hidden Host Mortality from an Introduced Parasitoid: Conventional and Molecular Evaluation of Non-Target Risk

**DOI:** 10.3390/insects11110822

**Published:** 2020-11-23

**Authors:** James R. Hepler, Kacie Athey, David Enicks, Paul K. Abram, Tara D. Gariepy, Elijah J. Talamas, Elizabeth Beers

**Affiliations:** 1 Tree Fruit Research and Extension Center, Washington State University, 1100 N Western Avenue, Wenatchee, WA 98801, USA; daencks@gmail.com (D.E.); ebeers@wsu.edu (E.B.); 2 Crop Sciences, University of Illinois at Urbana Champaign, 1101 W Peabody Drive, Urbana, IL 61801, USA; kathey@illinois.edu; 3 Agassiz Research and Development Centre, Agriculture and Agri-Food Canada, 6947 Highway 7, PO Box 1000, Agassiz, BC V0M 1A2, Canada; paul.abram@canada.ca; 4 London Research and Development Centre, Agriculture and Agri-Food Canada, 1391 Sandford Street, London, ON N5V 4T3, Canada; tara.gariepy@canada.ca; 5 Florida Department of Agriculture and Consumer Services, Division of Plant Industry—The Doyle Conner Building, 1911 SW 34th Street, Gainesville, FL 32614-7100, USA; Elijah.Talamas@FDACS.gov

**Keywords:** *Halyomorpha halys*, *Trissolcus japonicus*, non-target, hidden mortality, parasitoid, molecular forensics, nonreproductive effects, indirect effects

## Abstract

**Simple Summary:**

Classical biological control (CBC), i.e., the release of a natural enemy from the pest’s native range, has long been recognized as a viable approach to controlling invasive pests. CBC is considered specific to a given pest and does not come with the environmental risks often associated with broad-spectrum chemical pesticides. However, an exotic natural enemy may pose environmental risks, specifically to native species that are not the target of the biological control program. Some effects of an exotic natural enemy, such as consumption or reproduction, can be measured more readily than others. Our research points out that some of these ‘hidden effects’ can be quite important and merit our attention. We use molecular tools and modeling to help understand the hidden effects both for the target and non-target species. Our model system was an invasive fruit and vegetable pest from Asia, the brown marmorated stink bug (BMSB), *Halyomorpha halys*, and an exotic parasitoid, *Trissolcus japonicus*. *T. japonicus* has become established in North America and could help limit outbreaks of this pest. Unfortunately, it also kills non-target stink bugs with varying degrees of reproductive success, which may have both direct and indirect ecological effects on *H. halys* and non-target stink bug species.

**Abstract:**

Hidden trophic interactions are important in understanding food web ecology and evaluating the ecological risks and benefits associated with the introduction of exotic natural enemies in classical biological control programs. Although non-target risk is typically evaluated based on evidence of successful parasitism, parasitoid-induced host mortality not resulting in visible evidence of parasitism (i.e., nonreproductive effects) is often overlooked. The adventive establishment of *Trissolcus japonicus*, an exotic parasitoid of the introduced stink bug *Halyomorpha halys*, provides an opportunity to investigate the total impact of this parasitoid on target and non-target hosts in the field. We developed a new methodology to measure nonreproductive effects in this system, involving a species-specific diagnostic PCR assay for *T. japonicus*. We applied this methodology to field-deployed eggs of four pentatomid species, coupled with traditional rearing techniques. Nonreproductive effects were responsible for the mortality of an additional 5.6% of *H. halys* eggs due to *T. japonicus,* and were even more substantial in some of the non-target species (5.4–43.2%). The observed hidden mortality of native non-target species from an introduced parasitoid could change predictions about direct and indirect ecological interactions and the efficacy of biological control of the target pest.

## 1. Introduction

The accurate characterization of trophic interactions is important for several domains of applied ecology. A prime example is the classical biological control of invasive pests, in which natural enemies from the invasive species’ areas of origin are imported and released to provide long-term suppression in the areas of introduction [1]. Although many classical biological control introductions have been declared successful, the practice has come under heavy criticism in recent years due to a minority of cases in which introduced natural enemies have had negative impacts on non-target native species [2,3]. These concerns have led to a strict regulatory process in most countries, whereby thorough host range studies are required to assess the physiological and ecological host range of a biological control agent prior to approval for release [4,5]. Physiological host range studies determine the set of host species that can support the development of the natural enemy in the laboratory, whereas ecological host range studies determine the subset of physiologically compatible hosts that are actually utilized by the agent in the field. In both types of studies, the magnitudes of target and non-target effects are usually evaluated by measuring direct consumptive effects, which tend to be easily observed (e.g., a parasitoid emerges from a host, a predator consumes a prey). However, hidden interactions, which may occur when natural enemies attack a host (e.g., Condon et al. [6]), may be more common than is currently realized, and may be essential to understanding the ecological risk of introduced natural enemies as well as their broader role in trophic networks [7]. 

Parasitoid wasps are key components of terrestrial trophic networks [8]. The immature stages develop in or on the body of an insect host, and parasitism is considered successful when a host is killed as a result of offspring development, as evidenced by the emergence of a parasitoid adult. This particular component of host mortality is the easiest to observe and quantify and represents the impact of a parasitoid that contributes to its own reproduction (“reproductive effects”). However, there are a number of additional ways that parasitoids can reduce host populations which do not contribute to their own reproduction [7]. These “nonreproductive effects” constitute hidden trophic interactions, and include non-consumptive effects, lethal probing, and aborted parasitism [7]. Some of these effects can be measured relatively easily in the laboratory (e.g., by Abram et al. [9]), but their incidence in the field is much more challenging to detect and quantify. Traditional “collect, rear, and dissect” methods to assess parasitoid impact can only measure host death due to parasitism that is visually evident (i.e., the emergence of parasitoid adults or dissection of dead parasitoids from host tissues). However, some parasitoid-associated mortality may not be readily detected by visual examination of dead hosts, and therefore remains hidden when using traditional methodology to detect parasitoid activity [7,9]. In the context of classical biological control, this implies that the predicted occurrence of interactions between introduced agents and non-target hosts, and thus the severity of non-target effects of classical biological control, may be underestimated. 

The application of molecular forensics in ecology allows some previously hidden interactions to be uncovered, such as those that are lethal for consumers [6,10,11,12,13]. Further, molecular forensic approaches have been useful in detecting rare or unsuccessful lethal interactions, as well as in the identification of parasitoids or hosts following emergence [12,14,15,16,17]. The utility of molecular tools in this domain stems from their ability to detect trace amounts of host and parasitoid DNA, which makes them well-suited for the detection of parasitoid-induced host mortality in the absence of an identifiable adult. When applied in this context, novel molecular forensic data on nonreproductive effects may be useful in quantifying hidden interactions, and incorporating these data into non-target risk estimates may provide a more complete picture of the overall impact of a parasitoid in a trophic network.

*Halyomorpha halys* (Stål) (Hemiptera: Pentatomidae)*,* an Asian species, has become of global concern due to its establishment and rapid spread in several countries in Europe and North America [18]. A classical biological control program using *Trissolcus japonicus* (Ashmead) (Hymenoptera: Scelionidae), an Asian parasitoid, is being considered in most regions where the pest has become established or is likely to establish, and host range studies have been initiated in several countries to fulfill regulatory requirements for intentional releases. The adventive establishment of *T. japonicus* in several locations in the USA, Canada, and Europe was recently discovered [16,19,20,21,22]. This provides a unique opportunity to assess the impact of *T. japonicus* in the field and determine whether predictions made in pre-release host range studies were accurate. Further, it provides the opportunity to develop and test a new methodology that can be combined with the traditional methodology to estimate the impact of reproductive and nonreproductive effects of *T. japonicus* in the field. 

Previous molecular studies on pentatomid–scelionid associations used DNA barcoding to distinguish between multiple scelionid species that have overlapping geographic ranges. While DNA barcoding can provide informative and valuable data, particularly when the key players in the system are not well-described, it can be costly to sequence a large number of field-collected samples. Depending on the research question, this cost can be prohibitive, and a quicker, more affordable option, such as routine polymerase chain reaction (PCR) with species-specific primers, may be preferred if information on the presence or absence of a single species or a subset of species is required [13]. As *Trissolcus japonicus* is the species of interest in the present study, we developed a set of species-specific PCR primers for *T. japonicus* to allow rapid and cost-effective analysis of field samples. These primers were used in combination with standardized morphological assessment of sentinel host eggs to quantify reproductive and nonreproductive effects of *T. japonicus* on three native pentatomids (*Euschistus conspersus* (Uhler), *Chinavia hilaris* (Say), *Podisus maculiventris* (Say)), as well as *H. halys*, the most closely associated Asian host of *T. japonicus*. Using these results to parameterize existing theoretical models, we discuss how nonreproductive effects affect predictions of how direct and indirect ecological effects of introduced natural enemies contribute to biological pest control and non-target ecological impacts.

## 2. Materials and Methods

### 2.1. Primer Design

To design primers that amplify only DNA from *T. japonicus*, we collected all available cytochrome oxidase I (COI) sequences of *Trissolcus* spp. from GenBank. Sequences were aligned using MUSCLE [23] and visually inspected using BioEdit 7.0.0 (Isis Pharmaceuticals Inc., Carlsbad, CA, USA) to look for candidate 3′ locations. We used Primer3 [24] to optimize the primers. The primers were TJ-164F: (3′-TATTGTAACTTCACATGCATTTATTATAATC-5′) and TJ-395R: (3′-AAATTCCTGCTATATGTAGGGAAAAAATA-5′), producing a 231-bp amplicon. 

### 2.2. DNA Extraction and Optimization of Species-Specific PCR Assay

Total DNA (host and parasitoid, if present) was extracted using 100 μL of 5% Chelex solution and 2 μL of proteinase K. Dissected stink bug eggs were crushed with a flame sealed pipet tip as needed. The PCRs (12.5 µL) for primer design consisted of 1X Takara buffer (Takara Bio Inc., Shiga, Japan), 0.2 mM of each dNTP, 0.2 mM of each primer, 0.25 µL bovine serum albumin (Avantor, Radnor, Pennsylvania, USA), 1.25 U (Takara Ex Taq™, Takara, Mountain View, CA, USA) and template DNA (1 μL of total DNA). The PCRs were carried out in a Bio-Rad T100 thermal cycler (Bio-Rad Laboratories, Hercules, California, USA). The gradient PCR protocol was 94 °C for 1 min followed by 35 cycles of 94 °C for 20 s, 50–60 °C for 15 s, 72 °C for 15 s, and a final extension of 72 °C for 5 min. Each PCR included a positive and negative control. Following amplification, the products were visualized on 2% SeaKem agarose (Lonza, Rockland, ME, USA) pre-stained with GelRed nucleic acid gel stain (1X; Biotium, Hayward, CA, USA). The optimal annealing temperature was determined to be 57 °C. All PCR conditions were identical to those described above except the PCR cycling conditions were 94 °C for 1 min followed by 35 cycles of 94 °C for 20 s, 57 °C for 15 s, 72 °C for 15 s, and a final extension of 72 °C for 5 min. We then screened a set of non-target parasitoids in the genus *Trissolcus* including *T. cultratus* (Mayr), *T. euschisti* (Ashmead), *T. brochymenae* (Ashmead), *T. utahensis* (Ashmead)*, T. hullensis* (Harrington), and *T. edessae* (Fouts), as well as *Telenomus podisi* Ashmead. To ensure that cross-amplification with the pentatomid host egg did not occur, we also tested *H. halys, P. maculiventris, C. hilaris,* and *E. conspersus*. To ensure the broader applicability of the primers, we also screened *T. japonicus* strains originating from China (three strains), Switzerland, British Columbia, Canada, and the US states of Virginia, Ohio, and Utah. None of the non-target parasitoids or pentatomid samples produced a band when screened with our *T. japonicus* primers, and all the *T. japonicus* strains were positive for the primers. Any samples that did not produce a band were screened an additional time to reduce the risk of false negatives. 

### 2.3. Primer Sensitivity

Primer sensitivity was determined by testing dilutions of target DNA for amplification. The DNA concentration was determined using a NanoDrop 2000 Spectrophotometer (Thermo Fisher Scientific, Wilmington, DE, USA) adjusted to 5000 pg/µL and two-fold serially diluted. The serially diluted target DNA was used as a template at concentrations of 100, 50, 25, 12.5, 6.25, 3.13, 1.56, 0.78, 0.39, 0.20, 0.10, 0.05, and 0.025 pg/μL of target DNA.

### 2.4. Trissolcus japonicus Parasitism Time Series

A laboratory culture of *T. japonicus* was collected in 2018 from parasitized *H. halys* eggs in Vancouver, Washington. Adults were kept in 250 mL cups at 22 ± 3 °C, 55 ± 5% relative humidity, and a photoperiod of 16:8 (L:D). They were fed with a 50% honey solution, and the colony was perpetuated by periodic introduction of fresh (<24 h old) *H. halys* egg masses from a colony founded with wild adults collected from Washougal, Washington in 2019. The *H. halys* colony was maintained in 60 × 60 × 60 cm insect cages (Bug Dorm 6M-610, MegaView Science, Taichung City, Taiwan), under the same environmental conditions described above. They were fed a diet of lima bean plants (*Phaseolus lunatus* L. var. ‘Henderson’), organic carrots (*Daucus carota* L.), peanuts (*Arachis hypogaea* L.), pumpkin seeds (*Cucurbita pepo* L.), and sunflower seeds (*Helianthus annuus* L.). 

To create the parasitism time series, seven fresh *H. halys* egg masses on lima bean leaves were obtained from the colony. Each egg mass with a 3 mm margin of leaf tissue was placed in an arena (100 mL plastic cup) with one mated adult female *T. japonicus*. Females used in the test were 7 d old with no previous oviposition experience. Oviposition initiation was observed for each wasp, and the wasps and egg masses were together for 6 h. A control egg mass with no exposure to *T. japonicus* was also included. Following this, the adult female *T. japonicus* were frozen and the egg masses were held at 20 °C until their scheduled freezing time. The development time intervals were 0, 1, 2, 5, 7, or 10 days post oviposition (one egg mass/time point, 24.1 ± 0.6 eggs/mass). Following development time, the individual eggs were frozen at −20 °C for subsequent molecular analysis.

### 2.5. Field Exposure of Sentinel Egg Masses

We used sentinel egg masses from laboratory colonies of four stink bug species, including the main Asian host, *H. halys*, and three native North American species, to investigate the potential target and non-target effects of *T. japonicus* in the field. The *H. halys* colony was maintained as described above. Adults and nymphs of *E. conspersus* were collected from field sites in the mid-Columbia and central regions of Washington state. Adults of the green stink bug, *C. hilaris,* were shipped on ice packs from soybean fields in Kentucky in July 2019. The predatory stink bug *P. maculiventris* was obtained from a laboratory colony (Jeffrey R. Aldrich consulting, LLC, Marcell, MN, USA). The three phytophagous species were kept in 60 × 60 × 60 cm insect cages (Bug Dorm 6M-610, MegaView Science, Taichung City, Taiwan) in a controlled temperature room with ambient conditions and diet the same as described above for the *H. halys* colony. The bean plants served as a source of moisture and an oviposition substrate. Nymphs and adults were kept in separate cages to minimize cannibalism. *Podisus maculiventris* were kept in ventilated plastic boxes (32 × 25 × 10 cm) with mealworm pupae (*Tenebrio molitor* L.) (Rainbow Mealworms, Compton, CA, USA) for prey. Potted bean plants were included as a hydration source, and strips of heavy paper were placed in the rearing boxes for oviposition.

Egg masses <24 h old were removed from the colonies and mounted on a piece of cardstock (3 × 7 cm) using double-sided sticky tape. The egg masses were excised with a small piece of the oviposition substrate to retain any chemical cues left by the female during oviposition. Fine sand was sprinkled over the exposed tape to prevent entrapment of visiting parasitoids in the adhesive. After mounting and labeling, the egg masses were photographed, placed in a cooler, and transported to the field site. Field deployment was conducted from mid-July through late August on a *Paulownia* sp. tree in suburban Vancouver, WA, where a high level of *T. japonicus* was known to occur (45.63° N—122.67° W) [25]. Egg masses of *H. halys* were deployed on Tuesday and retrieved Friday of each week, while those of the native species were deployed on Fridays and collected Tuesdays; the exotic and native species eggs were never present at the same time. This temporal separation provided a more natural representation of how egg masses occur in the field, as opposed to a previous study by Milnes and Beers [25], in which egg masses belonging to the target and non-target host were deployed side-by-side (i.e., centimeters apart). The number of egg masses deployed on a given day varied depending on availability of eggs of the correct age and was limited by the reproductive output of the colonies. The strips of cardstock were attached with insect pins to the undersides of leaves scattered around the canopy (>1 m apart) ca. 1–3 m from the ground and exposed to parasitoids and predators for 3–4 d. 

Recovered egg masses were placed individually in 100 mm plastic Petri dishes and held at 20 °C to allow parasitoid development and emergence. After 6 weeks, adult parasitoids were removed, the egg masses re-photographed, and the images used to number each egg in the mass. The eggs were classified morphologically using the method of Morrison III et al. [26] for predation (codes C–G) and a modification of Waterworth et al. (unpublished) for emergence of the host nymph (code A) or parasitoid (code B) and the unhatched eggs (Table 1). Unhatched eggs (codes H–K) were dissected to further characterize the contents. Eggs were then placed individually in microcentrifuge tubes, retaining the egg species, replicate and egg number information, and held at −20 °C until PCR diagnosis. All eggs assigned to codes B–K were subjected to PCR analysis (see below). In addition, a subsample (up to two normally hatched eggs per mass) from code A (a stink bug nymph) were analyzed by PCR, although the fate of these eggs was considered unambiguous by morphological means. Adult wasps were pinned and identified by E.J.T. using the keys of Talamas [27] and Johnson [28]. 

### 2.6. PCR Diagnosis of Sentinel Eggs

The DNA from each egg was extracted using the Chelex protocol described above and screened with the species-specific *T. japonicus* PCR assay. In order to avoid false negatives and increase the detection of small quantities of DNA, all egg extractions were subjected to reamplification using 1 µL of PCR product from the first round of PCR as a template. This was repeated so each egg was screened using reamplification at least twice. Negative controls were included to check for cross-contamination. Reamplification bands were visualized on 2% SeaKem agarose (Lonza, Rockland, ME, USA) pre-stained with GelRed nucleic acid gel stain (1X; Biotium, Hayward, CA, USA). Reamplification could be particularly important to detect trace amounts of parasitoid DNA in empty eggs following parasitoid emergence (code B). Other studies have shown that empty parasitized eggs 7 d post-emergence have about a 50% amplification rate for parasitoid DNA [29]. This is congruent with our results for eggs of *H. halys* and *P. maculiventris* that were about 6 weeks post-emergence (55% PCR positive for *T. japonicus*). For *C. hilaris*, none of the empty parasitized eggs were positive for *T. japonicus*. However, our sample size was very low (*n* = 3). The relatively poor detection of parasitoid DNA in eggs from which the adult has eclosed is not unexpected, given that most of the DNA was removed with the adult. 

### 2.7. Data Analysis

For the purposes of analysis, we used the morphological characteristics combined with PCR results to group the data into reproductive (emerged adult parasitoid) and nonreproductive effects (Table 1). In the latter group, we included unemerged adult parasitoids (code H), all of which tested positive for *T. japonicus.* If the egg was PCR-positive, we included unemerged stink bugs (code I), unhatched eggs with no visible development (code J), and unhatched eggs with black residue (code K). This approach to estimating nonreproductive effects assumes that the presence of *T. japonicus* DNA in an egg indicates that the death of the egg was due to parasitism by *T. japonicus*. Because *T. japonicus* is an idiobiont parasitoid and kills its host during oviposition, this is a reasonable assumption unless some host eggs were already dead when deployed (see below). To account for this possibility, we developed approaches to calculate both upper and lower limits of nonreproductive effects to predict a reasonable range of uncertainty.

For a given stink bug species, an upper estimate of nonreproductive mortality, $NRE_{U}$, was calculated by summing the number of eggs in unascribed mortality categories associated with direct evidence of parasitism:

(1) $$NRE_{U}=N_{H}+N_{I\_pos}+N_{j\_pos}+N_{k\_pos}$$

where $N_{x}$ is the number of eggs in a given category x (*h*, *i*, *j*, or *k*). Recall that, in the case of category *h* (unemerged parasitoid adults), all eggs were included in the calculation whether PCR-positive or not. For categories *i*, *j*, and *k*, only PCR-positive eggs were considered to have been killed by nonreproductive effects. The number of PCR-positive eggs, $N_{x\_pos}$, was estimated for each category within each egg mass by summing eggs that tested positive, plus the estimated rate of PCR-positives for eggs that were untested, which was obtained by multiplying the proportion of tested PCR positive eggs in that category in the pooled dataset (for a given host species) by the number of untested eggs:

(2) $$N_{x\_pos}=N_{x\_tested\_pos}+\frac{N_{x_{untested}}N_{x\_total\_tested\_pos}}{N_{x\_total\_tested}}\;$$

Because $NRE_{U}$ assumes that every dead egg containing *T. japonicus* DNA was killed by *T. japonicus*, it risks overestimating nonreproductive effects if some eggs deployed in the field were already dead and were then subsequently attacked by *T. japonicus*. This parasitoid is known, for example, to readily attack infertile host eggs [30] as well as eggs that have been killed by freezing [31] and refrigeration [32]. Thus, we also calculated a lower estimate of nonreproductive effects, NRE_L_, which adjusts NRE_U_ to a lower value based on the mortality of PCR-negative eggs, i.e., it subtracts levels of baseline mortality from attacked eggs that would have occurred in the absence of parasitoid attack:

(3) $$NRE_{L}=NRE_{U}\left(1-\frac{N_{dead\_neg}}{N_{tot}}\right)\;$$

where $N_{tot}$ is the total number of eggs of each species placed in the field, and $N_{dead\_neg}$ is the sum of the estimated number of PCR-negative eggs in the three unascribed mortality categories (I, J, K), pooled across all egg masses placed in the field

(4) $$N_{dead\_neg}=N_{I\_neg}+N_{J\_neg}+N_{K\_neg}$$

The number of negative eggs for each category, $N_{x\_neg}$, was calculated as follows to include both the total number of tested negative eggs as well as the estimated number of negative untested eggs, based on the proportion of PCR-negative eggs (out of all that were PCR-tested):

(5) $$N_{x\_neg}=N_{x\_tested\_neg}+\frac{N_{x_{untested}}N_{x\_total\_tested\_neg}}{N_{x\_total\_tested}}$$

This approach assumes that the rate of baseline host egg mortality was the same in PCR-positive eggs as in PCR-negative eggs and that baseline mortality always preceded parasitism by *T. japonicus*. $NRE_{L}$ is a conservative estimate (i.e., it risks underestimating nonreproductive effects) because it assumes that *T. japonicus* is equally likely to attack viable and already-dead eggs. Because the correction uses pooled estimates, it also assumes the risk of *T. japonicus* attack is spread equally among egg masses.

Estimates of reproductive effects, $NRE_{U}$ and $NRE_{L}$, and total parasitoid-induced host mortality (hereafter, “impact”), with both upper and lower estimates of NRE, were analyzed with a generalized analysis of variance model with binomial distribution and logit link function (PROC GLIMMIX [33]). No random effect was added to the model. Species means were separated using least-squares means and the Tukey adjustment for experimentwise error. All data were analyzed as proportions (*n* reproductive, *n* nonreproductive, or *n* reproductive + nonreproductive/*n* total eggs in the mass). Additional calculations were made to summarize individual egg fates by species, and the proportion of egg masses with reproductive parasitism (i.e., where a parasitoid offspring was produced), and those with any evidence of parasitism. 

To test whether the odds of *T. japonicus* parasitism (=reproductive effects), nonreproductive effects, and egg mass discovery (i.e., whether each egg mass had any evidence of *T. japonicus* attack, either reproductive or nonreproductive effects) on non-target species is higher than for *Halyomorpha halys* at the egg mass level, we used resampling techniques (permutation tests and bootstrapping). For each non-target species, data were trimmed to include only weeks in which egg masses of both that non-target and *H. halys* were placed in the field to account for the possibility that parasitism pressure may have varied over time. Here, we also only used data from eggs that were subjected to PCR analysis, to avoid misattributing egg mass discovery due to undiagnosed eggs. The number of egg masses with *T. japonicus* parasitism and no parasitism for the non-target and *H. halys* were arranged in a 2 × 2 table. A permutation test (10^4^ replications) was used to generate a *p*-value as a test of independence. An odds ratio (i.e., the ratio of the odds of parasitism in the non-target compared to the odds of parasitism in *H. halys*) was calculated, and the 95% confidence interval on that odds ratio was calculated with bootstrapping (10^4^ bootstrap replications). Odds ratios more than 1 indicate the non-target species is more likely to be parasitized by *T. japonicus* than *H. halys*, whereas odds ratios less than 1 indicate the non-target species is less likely to be parasitized than *H. halys*. The process was then repeated to generate permutation test results and to calculate odds of non-target egg masses having *T. japonicus*-induced nonreproductive effects, relative to *H. halys* egg masses put out during the same time period. Finally, the process was repeated for the odds of discovery. 

### 2.8. Modeling of Direct and Indirect Ecological Effects of Nonreproductive Mortality

To generate predictions regarding the direct and indirect ecological effects of nonreproductive effects, we used the first model (“Model 1”) described in detail in Kaser et al. [34]. This model simulates a two-host, one parasitoid system and is based on difference equations outlined by Beddington [35] and further developed by Heimpel et al. [36], Kaser and Heimpel [37], and Abram et al. [9] to include the possibility of variation in host susceptibility and host mortality due to parasitism that does not result in parasitoid reproduction (parasitoid-induced host egg abortion, i.e., nonreproductive effects). We refer readers to Kaser et al. [34] for a detailed sensitivity analysis of this model. 

For all simulations in the current study, we held a number of parameters constant at values that favor model stability (see Heimpel et al. [36], Kaser and Heimpel [37], Abram et al. [9], Kaser et al. [34]). The carrying capacity of both hosts in the model (H1—the target species, *H. halys*; H2—the non-target species) was set at 100, and initial populations of both hosts were set to 20. The intrinsic rate of growth *r* for both host species was set at 0.5. The parasitoid aggregation parameter *k* was set at 0.75, and initial parasitoid populations were set at 5. These models assume randomly mixed host populations, which is unlikely to be the case for stink bugs in the field. However, some degree of population overlap is expected as both *H. halys* and the non-targets are habitat generalists and are known to co-occur under natural settings.

Based on what is known about the biology and ecology of scelionid egg parasitoids attacking *H. halys* and North American native stink bugs [9,38,39], parasitoid egg and/or time limitation was assumed, and so the parameter determining the degree of saturation of the parasitoids’ functional response (sometimes interpreted as egg load, but can also be interpreted as time limitation), β, was set at 5.

In each of the three simulations described below, we investigated the hypothesized direct and indirect population-level consequences of the levels of observed reproductive and non-reproductive effects measured in this study by varying three key parameters: attack rate, *a*; the proportion of parasitoid attacks resulting in parasitoid reproduction and host death, *s*; and the proportion of parasitoid attacks resulting in dead hosts but no parasitoid offspring emergence (nonreproductive effects), μ. To obtain equilibrium population levels of *H. halys* and non-target stink bugs from simulations with different parameter values (see below), simulations were run for 500 generations, after which stable equilibrium populations of hosts and parasitoids had always been reached.

First, we investigated the direct and indirect effects of including or not including the observed levels of nonreproductive effects on the target host *H. halys* and each of the non-target hosts in a two-host, one-parasitoid system. Parameters used in these simulations are shown in Table 2. First, we ran simulations that included the upper levels of nonreproductive effects (NRE_U_) observed in our study. *a* was set at a baseline value of 0.05 (based on sensitivity analyses in Kaser et al. [34]) for *H. halys* based on the total observed parasitoid impact in the presence of nonreproductive effects, and then scaled for each non-target based on the relative levels of parasitoid impact observed in our study). *s* was calculated based on the proportion of estimated parasitoid impact that was due to reproductive effects for each host species, and μ was set according to the proportion of total parasitoid impact that was due to nonreproductive effects, NRE_U_. Next, we ran the same simulations where the nonreproductive effects observed in our study were not included (i.e., simulating a situation where only successfully parasitism is measured, so it appears as if there are less parasitoid attacks, and they all result in successful parasitoid reproduction), downscaling *a* accordingly, setting all values of *s* to 1, and setting all values of μ to 0. Finally, to exclude indirect effects and examine only direct impacts of parasitism on each host, we ran each of the scenarios above for the target and non-target host in the absence of the other host by setting the other species’ initial population levels to zero. 

Second, to determine whether our model simulations would generate similar results if lower, conservative estimates of nonreproductive effects (NRE_L_) were used instead, we repeated the procedure above but instead used NRE_L_ to calculate *a*, s, and μ (Table 3). 

Third, to determine whether nonreproductive effects have unique predicted direct and indirect ecological consequences, we investigated whether adding the same amount of successful parasitism (reproductive effects) would generate similar predictions to the first set of simulations described above. Here, we used the same estimates of total parasitoid impact as in Table 2 but set *s* = 1 and μ = 0 (i.e., all parasitism events result in successful offspring production and host mortality) (Table 4). 

## 3. Results

### 3.1. Primer Design and Optimization of PCR Assay for T. japonicus

The *T. japonicus* species-specific PCR assay had 100% amplification success for *T. japonicus* adults and did not amplify DNA from the other parasitoid species tested (*T. cultratus, T. euschisti, T. utahensis, T. brochymenae, T. hullensis, T. edessae,* and *Telenomus podisi*), nor did it amplify DNA from any of the pentatomids tested. The absence of amplification of DNA from closely related species in the genus *Trissolcus* suggests these primers may be used to discriminate between the exotic and native parasitoids that we tested when no adult specimen is available. The sensitivity threshold of the *T. japonicus* PCR assay, as determined using serial dilutions, was 3.13 pg/μL of target DNA (without reamplification).

### 3.2. Trissolcus japonicus Parasitism Time Series

A total of 143 eggs exposed to *T. japonicus* was tested for parasitism, of which 139 tested positive for parasitoid DNA. The four that were negative were likely unparasitized. The assay successfully amplified *T. japonicus* DNA within *H. halys* eggs shortly after oviposition and throughout larval development. Of the egg masses exposed to *T. japonicus,* had 92–100% (all time points) of the eggs were positive for *T. japonicus* DNA, while the control *H. halys* egg mass had no amplification for *T. japonicus* DNA (Table 5). The eggs frozen immediately after the oviposition exposure (0 days, arguably the smallest amount of DNA) had 100% positive PCR results. All adult *T. japonicus* (positive controls) used in the test were positive when screened with the *T. japonicus* primer pair. 

### 3.3. Field Exposure and Diagnosis of Sentinel Egg Masses

**Figure 1:**
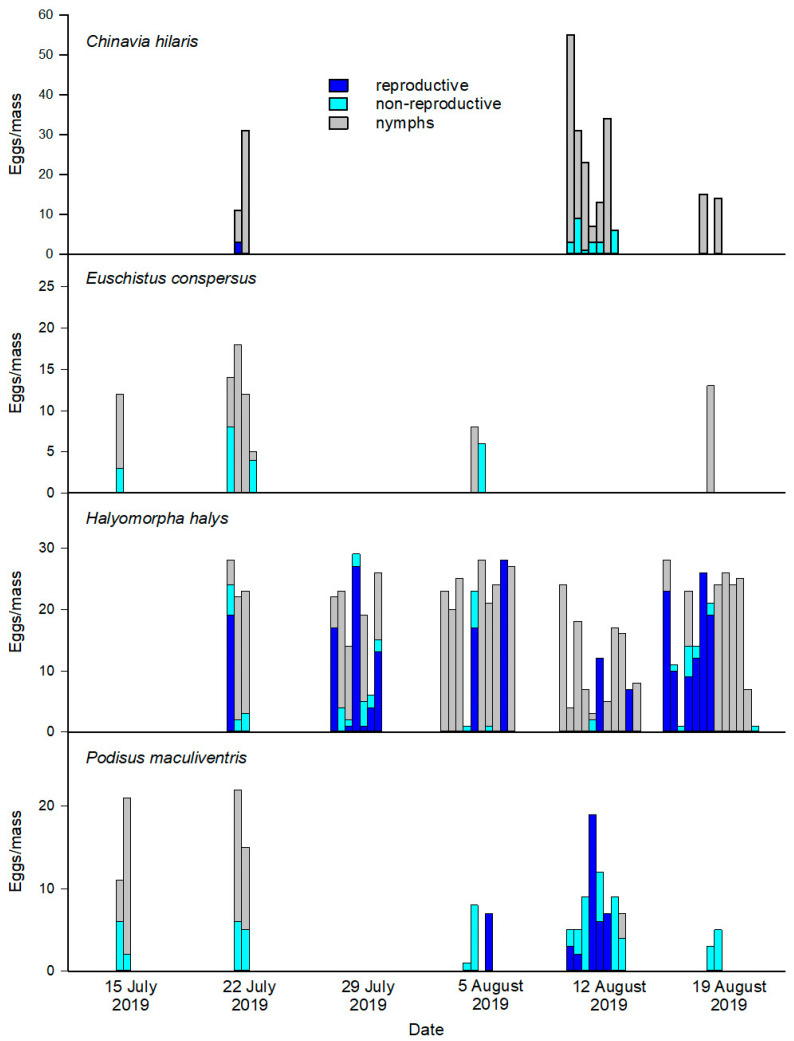
Occurrence of reproductive and nonreproductive effects during the study period. The colored portions in each bar represent the number of eggs in individual egg masses for each of four pentatomid species (*Chinavia hilaris, Euschistus conspersus, Halyomorpha halys,* and *Podisus maculiventris*) on a given date.

A total of 84 egg masses (1800 eggs) were evaluated using our combined classification scheme. Egg masses ranged in size by species: *C. hilaris* egg masses were the largest ($\bar{x}$ = 38.6 eggs ± SE 5.9) and most variable, followed by *H. halys* (22.7 ± 1.0), *E. conspersus* (13.8 ± 1.0), and *P. maculiventris* (12.2 ± 1.4). A total of 1013 eggs were evaluated using the *T. japonicus* PCR assay. Reproductive and nonreproductive parasitism occurred throughout the study period, with no apparent temporal pattern (Figure 1). All parasitoid adults emerging from exposed egg masses were identified by E.J.T. as *T. japonicus,* except for four adult *Anastatus reduvii* (Howard) that emerged from a single *E. conspersus* egg mass. This replicate was excluded from analyses. 

The egg fate outcomes varied by species (Table 6). Evidence of predation was low throughout the test (6 eggs) and occurred only in *E. conspersus* and *H. halys*. *Chinavia hilaris* had an unusually high percentage of unemerged nymphs (code I). However, only a small percentage of these were positive for parasitoid DNA using the PCR assay. Only seven of the unhatched eggs with no or partial development (codes J and K) were positive for *T. japonicus* DNA. While parasitoid-related mortality (reproductive and nonreproductive) was low, only 43.4% of the eggs hatched normally. *Euschistus conspersus* had a slightly higher successful hatch rate (59.8%, code A). The nonreproductive effects were most likely due to partially developed, unhatched eggs (codes J and K, averaging 63% PCR positive for *T. japonicus*). In the *H. halys* eggs, successful reproduction was the dominant form of parasitoid-induced mortality and 49.8% of the eggs hatched successfully. A few adult wasps did not emerge successfully (code H) and all of these were PCR positive. However, only 56.6% of the empty eggs with evidence of parasitoid emergence (code B) were PCR positive for *T. japonicus* DNA. Other unhatched eggs (codes I, J, and K) had low to moderate percentages of positive PCR results. Similar to *H. halys*, only 53.5% of *P. maculiventris* eggs from which parasitoids emerged were positive for *T. japonicus* DNA. Parasitoid activity was associated with 52.6 to 75.4% of the unhatched eggs, which collectively represented 54% of all eggs. Only 24.8% hatched successfully. 

**Figure 2:**
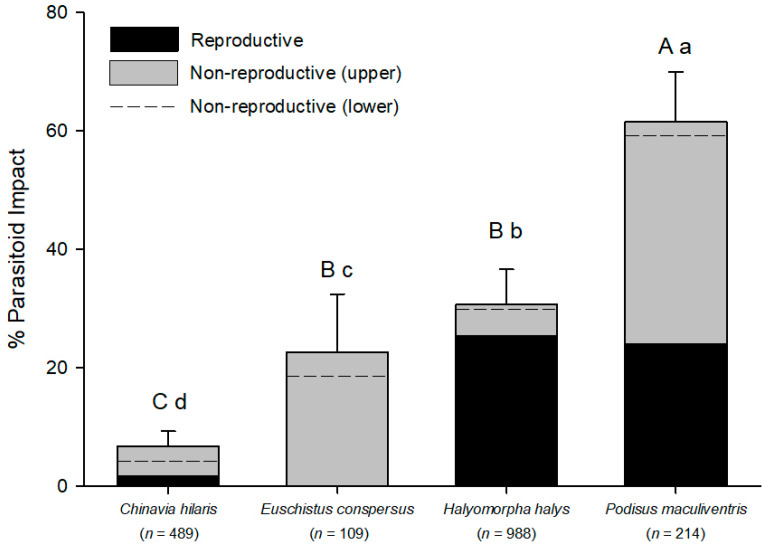
Reproductive and nonreproductive impacts of *T. japonicus* on four pentatomid host eggs. Reproductive effects: *F* = 15.79, *p* < 0.001; nonreproductive effects (upper estimate): *F* = 55.84, *p* < 0.001; nonreproductive effects (lower estimate): *F* = 48.15, *p* < 0.001; total impact (upper estimate of nonreproductive effects): *F* = 57.46, *p* < 0.001; total impact (lower estimate of nonreproductive effects): *F* = 49.65, *p* < 0.001; *df* all analyses 3, 80. Upper case letters above bars are for total impact (reproductive + nonreproductive) using the upper estimate of the nonreproductive effects, and lower case letters are for total impact using the lower estimate of nonreproductive effects. Numbers beneath the species names are the number of eggs evaluated. Error bars represent the standard error of the mean of total parasitoid impact (reproductive + nonreproductive).

Of the four pentatomid species tested, *C. hilaris* suffered the least amount of parasitoid impact (Figure 2). Reproductive impact (emerged adult *T. japonicus*) was 1.6%, caused by three adults from a single egg mass. Nonreproductive effects were 3.1 times higher than reproductive effects, and were observed in six egg masses. *Trissolcus japonicus* never successfully emerged from *E. conspersus* eggs, but nonreproductive impacts due to *T. japonicus* were observed in 22.7% of the *E. conspersus* eggs deployed. *Halyomorpha halys* had a total parasitoid impact of 30.7%. However, almost all of the effect occurred from successful parasitoid reproduction (25.5%). *Podisus maculiventris* eggs experienced the highest total parasitoid impact (61.6%). The nonreproductive effects of *T. japonicus* on *P. maculiventris* (37.6%) were 1.6-fold higher than the parasitoid’s reproductive effects on this host species (24.0%). 

The odds of a given egg mass being parasitized by *T. japonicus* (i.e., a parasitoid offspring emerged from at least one egg), affected by nonreproductive effects induced by *T. japonicus*, or being discovered by *T. japonicus* were similar among *H. halys* and two of the non-target species (*E. conspersus* and *C. hilaris*) tested (Table 7). For *P. maculiventris*, however, while the odds of egg mass parasitism were similar to *H. halys*, the odds of observing nonreproductive effects in a given *P. maculiventris* egg mass were more than 5 times higher than for *H. halys*. Combining instances of nonreproductive effects and successful parasitism, *P. maculiventris* egg masses were more than 16 times more likely to be discovered by *T. japonicus* than *H. halys* egg masses (Table 7). 

### 3.4. Modeling of Direct and Indirect Ecological Effects of Nonreproductive Mortality

**Figure 3:**
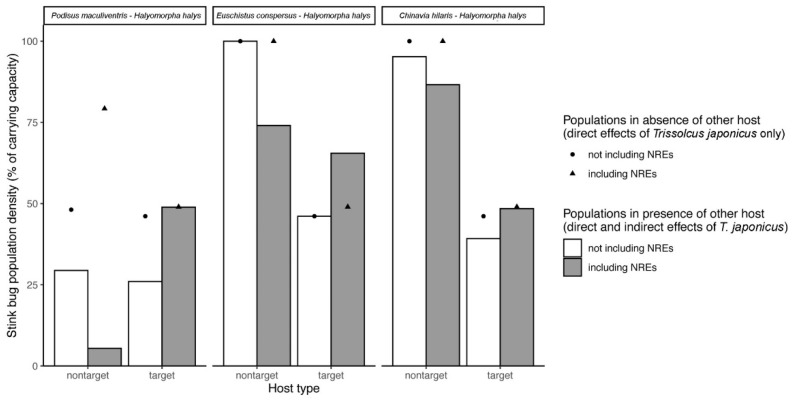
Simulated direct and indirect effects of *T. japonicus* on non-target and target stink bug populations in the absence and presence of nonreproductive effects (NREs), based on the population dynamics model of Kaser et al. (2018), and parameterized with data from the current study. See Table 2 for parameter values used. Upper estimates of nonreproductive effects (*NRE_U_*) was used, but similar results were obtained using lower estimates (NRE_L_) (Figure 4). Simulations were performed for three two-host, one-parasitoid systems consisting of *T. japonicus*, the target host *H. halys*, and one of three non-target species. For each host species pair, simulated equilibrium population densities of the non-target and target stink bug in the presence versus absence of measured nonreproductive effects, taking into account both direct and indirect (i.e., via the other host species) effects of *T. japonicus*, are shown as barplots. Simulated target and non-target population densities in the presence and absence of nonreproductive effects considering only direct effects of *T. japonicus* (i.e., in the absence of the other host species) are shown as points. When grey bars (including NERs) show values lower than paired white bars (not including NREs), the combined direct and indirect impact of nonreproductive effects in the system is to decrease stink bug population levels, and *vice versa*. When points are above bars, the host populations are being reduced as a consequence of the presence of the other host species in the system. When points are below bars, the host populations are higher as a result of the other host species being present in the system.

Results of the simulations examining the direct and indirect effects of non-reproductive mortality caused by *T. japonicus* in target (*H. halys*)–non-target species pairs are presented in Figure 3. These simulations indicate that taking the measured nonreproductive effects into account tends to increase estimated impacts of *T. japonicus* on non-target stink bugs and decreases its biological control effect on *H. halys* (Figure 3). This general outcome can be explained through the combined contributions of both direct and indirect ecological effects. Taking nonreproductive effects into account, the predicted direct effect in the long term is to decrease the proportional impact of *T. japonicus* on non-target stink bugs and *H. halys* because it lowers parasitoid population levels (and thus their impact on hosts) by decreasing the proportion of attacks that produce offspring. Indirect effects, on the other hand, are completely responsible for the hypothetical net equilibrium population increases of *H. halys* and decreases in those of native stink bugs in the presence of nonreproductive effects. When nonreproductive effects are not considered, both *H. halys* and non-target stink bugs are predicted to experience apparent competition; i.e., the presence of another host in the environment for *T. japonicus* increases population levels of the parasitoid and drives both target and non-host population levels down (relative to if the other host was absent). Adding nonreproductive effects, which occur more on non-target stink bugs, disproportionately reduces the intensity of apparent competition affecting *H. halys*. This is because parasitoids would be devoting time and eggs (i.e., saturating their functional response) attacking the non-target stink bugs that do not contribute to increasing the parasitoid population. The degree to which *H. halys* experiences relaxed apparent competition depends on the prevalence of nonreproductive effects in the non-target species. When the proportion of non-target attacks that result in nonreproductive effects is the highest (*E. conspersus*), the predicted indirect ecological effect of the non-target switches from apparent competition to apparent predation. That is, the presence of *E. conspersus* overall benefits *H. halys* because *T. japonicus* is wasting eggs and time on this mostly unsuitable host, reducing its impact on the more suitable host (Figure 3). For *P. maculiventris* and *C. hilaris,* the net result of including nonreproductive effects is to change the predicted interaction from apparent competition (both hosts suffer from each other’s presence) to apparent amensalism (*H. halys* negatively impacts non-target populations, but there is no effect of non-target species on *H. halys* populations).

**Figure 4:**
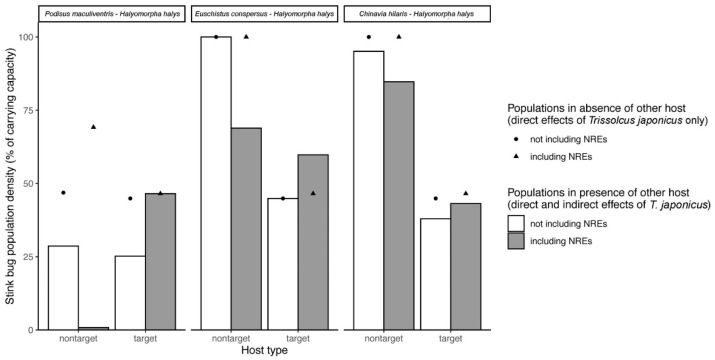
Simulated direct and indirect effects of *T. japonicus* on non-target and target stink bug populations in the absence and presence of nonreproductive effects (NREs), using lower estimates (NREL). See Table 3 for parameter values used. Simulations were done for three two-host, one-parasitoid systems consisting of *T. japonicus*, the target host *H. halys*, and one of three non-target species. For each host species pair, simulated equilibrium population densities of the non-target and target stink bug in the presence versus absence of measured non-reproductive effects, taking into account both direct and indirect (i.e., via the other host species) effects of *T. japonicus*, are shown as barplots. Simulated target and non-target population densities in the presence and absence of non-reproductive effects considering only direct effects of *T. japonicus* (i.e., in the absence of the other host species) are shown as points. When grey bars show values lower than paired white bars, the combined direct and indirect impact of non-reproductive effects in the system is to decrease stink bug population levels, and *vice versa*. When points are above bars, the host populations are being reduced as a consequence of the presence of the other host species in the system. When points are below bars, the host populations are higher as a result of the other host species being present in the system.

Results of the simulations using lower estimates of nonreproductive effects (*NRE_L_*) are presented in Figure 4. Simulation results using *NRE*_L_ were qualitatively similar to those using *NRE*_U_ (see Figure 3) in terms of the direction of the direct and indirect effects of including nonreproductive effects on target and non-target population levels. There were some small differences due to the fact that when *NRE*_L_ was used, a lower proportion of parasitoid ovipositions resulted in successful offspring production. This led to a weakening of apparent competition effects, resulting in overall higher non-target stink bug populations (e.g., *P. maculiventris*) when *NRE_L_* nonreproductive effects were considered (relative to using *NRE_U_*) (Figure 4). In the *H. halys*–*E. conspersus* system, using *NRE_L_* (Figure 4) instead of *NRE_U_* (Figure 3) results in a slightly weaker apparent predation effect.

**Figure 5:**
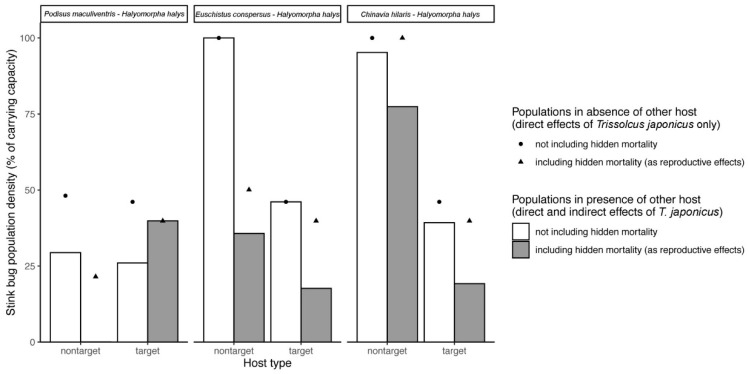
Simulated direct and indirect effects of *T. japonicus* on non-target and target stink bug populations if hidden host mortality (due to nonreproductive effects, NREs) is excluded, or is included as attack resulting in parasitoid reproduction (reproductive effects) (to contrast with Figure 2 above where the same amount of mortality is added as nonreproductive effects). See Table 4 for parameter values used. Simulations were done for three two-host, one-parasitoid systems consisting of *T. japonicus*, the target host *H. halys*, and one of three non-target species. For each host species pair, simulated equilibrium population densities of the non-target and target stink bug in the presence versus absence of additional mortality due to parasitism, taking into account both direct and indirect (i.e., via the other host species) effects of *T. japonicus*, are shown as barplots. Simulated target and non-target population densities in the presence and absence of additional mortality due to parasitism considering only direct effects of *T. japonicus* (i.e., in the absence of the other host species) are shown as points. When grey bars show values lower than paired white bars, the combined direct and indirect impact of additional host mortality in the system is to decrease stink bug population levels, and *vice versa*. When points are above bars, the host populations are being reduced as a consequence of the presence of the other host species in the system. When points are below bars, the host populations are higher as a result of the other host species being present in the system.

Finally, simulations where additional mortality (measured as non-reproductive effects) was instead added to the models as reproductive effects (Figure 5) qualitatively differed from results that include additional mortality as non-reproductive effects. First, in general, there were greater direct negative effects of additional parasitoid-induced mortality on both non-targets and targets. Second, combined direct and indirect effects of additional mortality tended to increase parasitoid impact on the target host, because of more intense apparent competition resulting from more successful parasitoid reproduction. For example, the apparent predation interaction in the *E. conspersus–H. halys* system would change to apparent competition. The exception is in the *P. maculiventris–H. halys* pair; in this parameter space, simulated *P. maculiventris* populations went extinct under the scenario of higher mortality due to successful parasitism and thus had no apparent competition effect on *H. halys* (Figure 5).

## 4. Discussion

The study of exotic biological control agents following their establishment allows the host range and impact of the agent to be evaluated in the area of introduction, and provides the opportunity to determine whether pre-release host range studies adequately predicted non-target risk [4,40]. Such information can be used to refine existing risk assessment methodology and guide future release or control programs. The adventive establishment of *T. japonicus* in the North American and European areas of invasion of *H. halys* [19,20] presents multiple opportunities to conduct retrospective analyses of the proposed biological control introduction, allowing the direct and indirect ecological impacts of the parasitoid on the target and non-target pentatomid population to be evaluated. Our results, in line with laboratory results, suggest that adventive *T. japonicus* do in fact attack non-target North American pentatomids in the field and that levels of successful parasitism can vary substantially among species. As we discuss below, the considerable amount of unsuccessful parasitoid attack causing host death that we detected in this study has the potential to have both direct and indirect ecological effects in parasitoid-pentatomid trophic networks.

An underestimation of parasitoid impact on non-target hosts is a major concern in classical biological control [10]. However, molecular approaches can quantify parasitism that may be underestimated due to high levels of host mortality in traditional rearing [41]. Mortality due to nonreproductive effects may be more severe when a host and parasitoid do not share an evolutionary history (as is the case in classical biological control), as physiological incompatibilities may prevent successful parasitoid development [34,38]. Detection of DNA from dead, undeveloped *Trissolcus* eggs and larvae in an incompatible pentatomid host has been demonstrated previously [42]. Our results are consistent with these past findings and confirm that trace amounts of parasitoid DNA are detectable regardless of the developmental outcome in an unsuitable host. Thus, we implemented molecular forensics in the post-establishment evaluation of the exotic parasitoid *T. japonicus* on target and non-target hosts under field conditions. Our results demonstrated that molecular tools are able to detect additional attacks that would not be apparent with traditional techniques and show that nonreproductive effects are occurring in this biological control system, although their prevalence varies among host species. 

The two native phytophagous pentatomids in our study (*C. hilaris* and *E. conspersus*) had lower levels of successful egg parasitism by *T. japonicus* than did *H. halys*, a finding consistent with the previous field studies at the same location [25]. In the present study, *C. hilaris* also appeared to have low levels of nonreproductive effects, as indicated by a low number of unemerged parasitoids and a low number of unhatched eggs testing positive for *T. japonicus* DNA. However, an abnormally high level of unemerged *C. hilaris* nymphs (50%, code I) occurred in the present study, and most were negative for *T. japonicus* DNA. It is possible that some of these unemerged nymphs were the result of natural (not explained by predation or parasitism) egg mortality. In fact, laboratory host range studies on a closely related species, *Nezara viridula* (both species are in the tribe Nezarini), found similar levels of unemerged nymphs (approx. 50%) in egg masses that were exposed to *T. japonicus* [43,44], and unexposed, control egg masses of *Nezara viridula* also had approximately 50% unemerged eggs [44,45], indicating that the high levels of mortality in this group may be unrelated to parasitoid exposure. However, unemerged eggs could also, at least in principle, be as a result of unsuccessful oviposition by a parasitoid (i.e., probing) that does not leave DNA evidence. Laboratory studies have shown that some members in the tribe Nezarini are poor reproductive hosts for *T. japonicus* [20,43,44,45], and higher levels of unexplained mortality in *T. japonicus*-exposed versus unexposed eggs have been observed in some studies [45]. The detection of parasitoid DNA from probing without oviposition has not been tested but is unlikely to result in detectable levels of parasitoid DNA unless other substances (e.g., venom, arrestment factors) are injected by the female parasitoid. If probing alone causes high levels of mortality in some species, then it is possible that a portion of nonreproductive effects may be overlooked even by molecular diagnostics, but this remains to be tested. Recent studies have documented features of oviposition marks on *H. halys* eggs that appear to be diagnostic at the family level [46]. Detection and quantification of these marks on eggs that have no parasitoid DNA may yield further insight into probing as a source of nonreproductive mortality. In future studies it would also be prudent to expose control egg masses to the same environmental field conditions in a way that excludes parasitoid access to the egg mass (i.e., using fine mesh cages or sleeves) to provide a more accurate assessment of natural mortality. In addition to significantly higher mortality levels, laboratory studies by Haye et al. [45] also observed a significantly longer handling time of *N. viridula* egg masses in comparison to *H. halys*, even when egg mass size was controlled. It would be interesting to determine whether this also occurs in *Chinavia* (and other Nezarini) and whether increased probing is correlated with higher host mortality. It also supports the concept that risk assessment studies should move beyond “black box” approaches and incorporate direct observation to determine the relationship between parasitoid behavior and development [45].

The mortality described above for *C. hilaris* was not widespread across the species investigated here. In fact, although *E. conspersus* had similar levels of unhatched, partially developed eggs in the present study and in Milnes and Beers [25], PCR diagnosis suggests that the majority of unhatched eggs were the result of nonreproductive effects from *T. japonicus*. Laboratory host range studies on *Euschistus* spp. generally show no acceptance [20] or low acceptance and low emergence of *T. japonicus* from this non-target host [47], with a similar percentage of unemerged or dead eggs (~25–40%) as we found in the present study (36%). The combined effects of *T. japonicus* on *E. conspersus* led to a mortality level similar to that experienced by *H. halys*, but it was primarily due to nonreproductive effects in *E. conspersus*, while the mortality was due to reproductive effects in *H. halys*.

The total impact of *T. japonicus* on *P. maculiventris* in field-exposed sentinel egg masses was higher than expected. The combined reproductive and nonreproductive mortality of 62% in field-exposed sentinel egg masses was approximately 2× higher than on the target host, *H. halys*. Previously conducted laboratory host range studies have predicted non-target parasitism on *P. maculiventris*, but results varied in terms of the level of parasitoid-induced mortality. Lara et al. [43] observed the highest levels of successful parasitism of *P. maculiventris* (56% in no-choice tests and 41% in choice tests), while Botch and Delfosse [47] reported 13–27% parasitism (depending on the natal host species) in multiple-species choice tests. Hedstrom et al. [20] reported 13% mean parasitism of *P. maculiventris* by *T. japonicus* in no-choice tests. The high variability among these results makes it difficult to interpret, but nonetheless, they state that *P. maculiventris* was never successfully parasitized. Interestingly, these studies indicate that a considerable number of eggs (ranging from 20–40%) failed to yield either a parasitoid adult or a stink bug nymph, indicating that unexplained mortality was present. Based on our results, it seems likely that the majority of this mortality was due to nonreproductive effects, which, if added to their observed parasitism level, would demonstrate a total impact (from both reproductive and nonreproductive effect) similar to the one demonstrated in our study. 

As previously undetected nonreproductive effects are uncovered in host-parasitoid trophic networks, the next challenge is to understand how they translate to population-level consequences in order to weigh risks versus benefits in a biological control program. The most easily understood population-level impacts of nonreproductive effects are the direct ecological effects, in this case, to what degree the additional non-target mortality due to nonreproductive effects by *T. japonicus* directly translate to lower populations of non-target stink bugs. Modified indirect ecological effects on both non-target stink bugs and *H. halys*, mediated by their shared parasitoid, are a second potential consequence of the observed nonreproductive effects. Indirect ecological effects, the best known of which is apparent competition, are of increasing interest in biological control [36,37,48] due to their potential to both affect the level of non-target risk and the efficacy of control of the target pest. Kaser et al. [34], using *H. halys* and its parasitoids as a case study, developed a two-host, one-parasitoid difference equation population dynamics model to predict the direction and relative strength of direct and indirect ecological effects of nonreproductive effects. Here, we parameterized this model with the proportion of reproductive and nonreproductive non-target mortality due to *T. japonicus* found for each of the non-target species in the current study (Figure 3). This allowed us to generate new hypotheses about how nonreproductive effects might drive non-target risk and biological control efficacy in this system via both direct and indirect ecological effects in simplified *T. japonicus*-*H. halys*-non-target food webs. 

**Figure 6:**
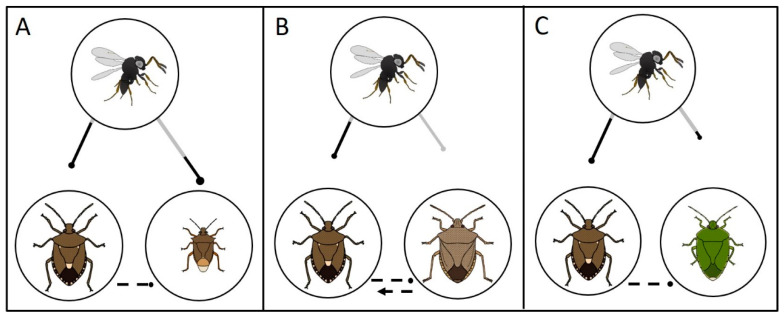
Modified Levins’ Diagrams showing examples of hypothesized indirect and direct effects of *Trissolcus japonicus* on target and non-target stink bugs, and indirect interactions between stink bug species pairs, based on predictions from population dynamics models (see Figure 3). Dots at the end of lines indicate a negative effect on the stink bug species shown; arrows indicate a positive effect. Solid lines are direct effects and dashed lines are indirect effects. For all effects of *T. japonicus* on stink bugs, the length of the line indicates magnitude of the interaction. The gray portion of the line corresponds to the nonreproductive contributions and the black portion corresponds to the reproductive effect. Where the dotted line is missing in one direction, no indirect effect was observed. Effects of stink bugs on *T. japonicus* populations were not evaluated in this study, and are not shown here. (**A**) *Halyomorpha halys* (target) and *Podisus maculiventris* (non-target); apparent amensalism. (**B**) *Halyomorpha halys* (target) and *Euschistus conspersus* (non-target); apparent predation. (**C**) *Halyomorpha halys* (target) and *Chinavia hilaris* (non-target); apparent amensalism.

Based on our results and model simulations (see Section 3.4), it is predicted that *H. halys* biocontrol with *T. japonicus* could sometimes be less effective when non-target stink bug species are present. This may especially be the case for non-target species such as *E. conspersus* that experience high levels of unsuccessful parasitoid attack and thus act as an “egg sink” that indirectly benefits *H. halys* [9,34,36]. In addition, the non-target impacts of *T. japonicus* on native stink bugs are always predicted to be greater when *H. halys* is present, and the magnitude of this effect will be highest when non-target species are attacked at relatively high rates (e.g., *P. maculiventris*). Predicted indirect ecological effects due to nonreproductive parasitoid attack of non-target species are summarized in Figure 6. The ongoing establishment of *T. japonicus* in environments with *H. halys* co-occurring with non-target stink bug species across the world provides an opportunity to test these hypotheses in the coming years, while broadening the scope of studies to understand the role of potential spatial (habitat) and temporal refugia from parasitism [49].

The analysis of ecological interaction networks has enormous potential for improving predictions about the direct and indirect ecological consequences of both intentional and unintentional biological control introductions [50]. If hidden lethal interactions such as those described in this study are common (see also Condon et al. [6]), it suggests that current methods used to analyze trophic networks could benefit from refinement to incorporate information not only the existence and strength of links between network nodes, but also how the nature of these links could differ in terms of how they affect consumer versus resource population levels (e.g., Figure 6). Current methodologies for establishing the presence of links between nodes in trophic host-parasitoid networks used to predict ecological outcomes [50,51,52] consider whether hosts are attacked by parasitoids or not, and sometimes at what frequency. However, to our knowledge they typically do not distinguish whether the attack results in the death of the host, death of the parasitoid, or death of both—despite the hypothetical implications for host-parasitoid population dynamics that we have demonstrated in the current study. Simply increasing the overall interaction strength by adding measured nonreproductive effects is not sufficient. According to our simulations, this approach generates markedly different predictions about direct and indirect effects in food webs because the hosts that die as a result of nonreproductive effects do not contribute to increasing parasitoid populations (Section 3.4; Figure 3 and Figure 5). Thus, further theoretical development may be needed to incorporate these types of interactions into existing network analyses in certain biological control systems where they have been shown to be prominent. How data are collected to construct trophic networks will also be fundamental to making proper inferences. As illustrated by our study, conventional assessments of parasitism tend to overlook interactions that do not result in parasitoid emergence, while molecular methods only distinguish whether an interaction occurred, but not whether a parasitoid would emerge or the host would die as a result. Incorporating these hidden lethal effects into trophic network analyses will thus require a continued pairing of traditional and molecular techniques.

Field studies using naturally-occurring or sentinel hosts in a variety of crop and non-crop habitats would be useful to determine whether non-target hosts have access to spatial or temporal refuges where they are not attacked as frequently or efficiently by the introduced parasitoid [40]. These studies could provide insight into the magnitude of non-target effects and determine whether there is the potential for *T. japonicus* to cause significant and persistent mortality in non-target pentatomid populations. Any significant, long-term population reductions would be particularly undesirable in the case of beneficial predatory species such as *P. maculiventris.* Studies in China, Europe, and New Zealand on the parasitism of other species of predatory Asopinae by *T. japonicus* typically also reported successful parasitism of >90% of egg masses in no-choice tests, with no significant difference in parasitism of the target host (*H. halys*) and the non-target Asopinae [*Arma chinensis* Fallou [53], *Arma custos* (Fab.) [45], and *Cermatalus nasalis* (Westwood) [44]]. This supports our finding that predatory Asopinae have the potential to be heavily exploited by this parasitoid. However, the extent of this exploitation and the population-level impact needs to be explored further, particularly in light of the magnitude of nonreproductive effects that we have demonstrated in the current study. In addition, the likelihood of reduced fitness of *T. japonicus* that develop in predatory Asopinae [47] may have consequences in terms of the sustainability of a parasitoid population in a non-target host over time. 

## 5. Conclusions

Although this study was limited to a single host plant and a single site with high *T. japonicus* activity, it serves as a proof-of-concept to develop and test the utility of this approach, which can be applied to future, ongoing studies that seek to address the benefits and risks associated with the establishment of *T. japonicus* for *H. halys* control. While the hazard component of risk appears to be high, our study did not provide adequate information on exposure (see Delfosse [51,54]). To further validate this approach, the application of this tool to larger scale field studies will be necessary to determine the impact of *T. japonicus* on non-target host populations in different habitats throughout the season. Overall, our results clearly demonstrate that the direct and indirect ecological influence of nonreproductive effects should not be ignored in parasitoid–pentatomid associations, at the risk of greatly underestimating the non-target effects of exotic parasitoids in the field.

## Figures and Tables

**Table 1:** Egg fates used for classifying outcomes after exposure to parasitoids in the field.

Fate Code	Description	Diagnosis Type	Effect Type Group
A	Stink bug nymph	Morphological	Other (normal development)
B	Emerged parasitoid adult	Morphological	Reproductive
C	Complete chew	Morphological	Other (predation)
D	Incomplete chew	Morphological	Other (predation)
E	Stylet sheath	Morphological	Other (predation)
F	Punctured, but no stylet sheath (spider)	Morphological	Other (predation)
G	Host Feeding (*Anastatus*)	Morphological	Other (predation)
H	Unemerged adult parasitoid	Morphological	Nonreproductive
I	Unemerged stink bug	PCR	Nonreproductive (PCR dependent)
J	Unhatched egg, no development	PCR	Nonreproductive (PCR dependent)
K	Unhatched egg, black residue	PCR	Nonreproductive (PCR dependent)

**Table 2:** Parameters used in population simulations determining the effect of including the upper estimate of non-reproductive effects of *T. japonicus* on *H. halys* and three species of non-target hosts. Detailed information regarding model parameters can be found in Section 2.8.

	With NRE_U_ Included			Without NRE_U_ Included		
Host Species	Attack Rate, *a*	Susceptibility, *s*	Egg Abortion, µ	Attack Rate, *a*	Susceptibility, *s*	Egg Abortion, µ
*Halyomorpha halys*	0.050	0.821	0.179	0.041	1.000	0.000
*Podisus maculiventris*	0.108	0.357	0.643	0.039	1.000	0.000
*Euschistus conspersus*	0.037	0.000	1.000	0.000	1.000	0.000
*Chinavia hilaris*	0.011	0.233	0.767	0.003	1.000	0.000

**Table 3:** Parameters used in population simulations determining the effect of including the lower estimate of non-reproductive effects of *T. japonicus* on *H. halys* and three species of non-target hosts. Detailed information regarding model parameters can be found in Section 2.8.

	With NRE_L_ Included			Without NRE_L_ Considered		
Host Species	Attack Rate, *a*	Susceptibility, s	Egg Abortion, µ	Attack Rate, *a*	Susceptibility, *s*	Egg Abortion, µ
*Halyomorpha halys*	0.050	0.851	0.186	0.043	1.000	0.000
*Podisus maculiventris*	0.099	0.405	0.728	0.040	1.000	0.000
*Euschistus conspersus*	0.031	0.000	1.211	0.000	1.000	0.000
*Chinavia hilaris*	0.007	0.381	1.253	0.003	1.000	0.000

**Table 4:** Parameters used in population simulations determining the effect of including additional reproductive mortality equal to the value of non-reproductive effects of *T. japonicus* on *H. halys* on three species of non-target hosts. Detailed information regarding model parameters can be found in Section 2.8.

	With Additional Mortality Considered			Without Additional Mortality Considered		
Host Species	Attack Rate, *a*	Susceptibility, *s*	Egg Abortion, µ	Attack Rate, *a*	Susceptibility, s	Egg Abortion, µ
*Halyomorpha halys*	0.050	1	0	0.041	1	0
*Podisus maculiventris*	0.108	1	0	0.039	1	0
*Euschistus conspersus*	0.037	1	0	0.000	1	0
*Chinavia hilaris*	0.011	1	0	0.003	1	0

**Table 5:** PCR-amplified *Halyomorpha halys* eggs exposed to *Trissolcus japonicus* females using species-specific *T. japonicus* primers.

Time	Number of Eggs Tested	Number of Eggs Amplified	% Amplified
0 days, unparasitized	26	0	0
0 days	23	23	100
1 day	23	22	96
2 days	22	21	95
5 days	25	25	100
7 days	24	24	100
10 days	26	24	92

**Table 6:** Egg fates and PCR outcomes of sentinel egg masses of four pentatomid species.

Species	Fate	Fate Name	*n* Eggs Evaluated	*n* Eggs PCR	*n* Eggs PCR Positive	% PCR Positive	% of Total in Category
*Chinavia hilaris*	A	Stink bug nymph	212	17	2	12	43.40%
	B	Emerged parasitoid adult	3	3	0	0	0.60%
	H	Unemerged adult parasitoid	5	5	5	100	1.00%
	I	Unemerged stink bug	246	243	13	0	50.30%
	J	Unhatched egg, no development	21	17	6	35	4.30%
	K	Unhatched egg, black residue	2	2	1	50	0.40%
		Sum	489	287	27		100%
*Euschistus conspersus*	A	Stink bug nymph	67	13	0	0	61.50%
	D	Incomplete chew	2	2	0	0	1.80%
	J	Unhatched egg, no development	26	26	9	35	23.90%
	K	Unhatched egg, black residue	14	14	12	86	12.80%
		Sum	109	55	21		100%
*Halyomorpha halys*	A	Stink bug nymph	492	66	3	5	49.80%
	B	Emerged parasitoid adult	245	235	133	57	24.80%
	D	Incomplete chew	2	2	0	0	0.20%
	E	Stylet sheath	2	2	0	0	0.20%
	H	Unemerged adult parasitoid	8	7	7	100	0.80%
	I	Unemerged stink bug	88	84	7	8	8.90%
	J	Unhatched egg, no development	88	85	11	13	8.90%
	K	Unhatched egg, black residue	63	59	21	36	6.40%
		Sum	988	540	182		100%
*Podisus maculiventris*	A	Stink bug nymph	53	9	0	0	24.80%
	B	Emerged parasitoid adult	44	43	23	53	20.60%
	I	Unemerged stink bug	29	28	16	57	13.60%
	J	Unhatched egg, no development	66	57	43	75	30.80%
	K	Unhatched egg, black residue	22	19	10	53	10.30%
		Sum	214	156	92		100%

**Table 7:** Frequency of three outcomes for the subset of non-target egg masses that was placed in the field during the same weeks as *H. halys* egg masses: parasitism (reproductive effects), nonreproductive effects, and discovery (parasitism and/or nonreproductive effects). Percentages indicate the percent of non-target egg masses with at least one egg with relevant outcome. Asterisks indicate that the frequency of a given outcome for non-target egg masses is significantly different than for time-matched *H. halys* egg masses, as per permutation tests (* *p* < 0.01; no symbol, *p* > 0.05); Odds ratios (OR), with 95% confidence intervals (CIs) show the ratio of odds of each outcome for non-target egg masses relative to time-matched *H. halys* egg masses. Odds ratios greater than 1 indicate a higher odds of the outcome for the non-target species than *H. halys*; odds ratios less than 1 indicate lower odds of the outcome for the non-target species.

Non-Target Species (*n* Non-Target Egg Masses, *n* Time-Matched *H. halys* Egg Masses)	Parasitism		Nonreproductive Effects		Discovery	
	Percentage	OR (95% CI)	Percentage	OR (95% CI)	Percentage	OR (95% CI)
*Podisus maculiventris* (*n* = 16, 38)	37.5%	1.47 (0.35–5.33)	75.0% *	5.77 (1.76–32.5)	93.8% *	16.67 (3.31–28.85)
*Euschistus conspersus* (*n* = 7, 27)	0%	0.00 (0.00–0.00)	42.9%	0.60 (0.07–3.64)	42.9%	0.60 (0.07–3.64)
*Chinavia hilaris* (*n* = 13, 28)	7.7%	0.17 (0.00–1.09)	46.2%	1.54 (0.35–6.76)	53.8%	1.17 (0.28–5.15)
